# Soap-film coating: High-speed deposition of multilayer nanofilms

**DOI:** 10.1038/srep01477

**Published:** 2013-03-18

**Authors:** Renyun Zhang, Henrik A. Andersson, Mattias Andersson, Britta Andres, Håkan Edlund, Per Edström, Sverker Edvardsson, Sven Forsberg, Magnus Hummelgård, Niklas Johansson, Kristoffer Karlsson, Hans-Erik Nilsson, Magnus Norgren, Martin Olsen, Tetsu Uesaka, Thomas Öhlund, Håkan Olin

**Affiliations:** 1 Department of Applied Science and Design, Mid Sweden University, SE-85170 Sundsvall, Sweden; 2 Department of Information Technology and Media, Mid Sweden University, SE-85170 Sundsvall, Sweden

## Abstract

The coating of thin films is applied in numerous fields and many methods are employed for the deposition of these films. Some coating techniques may deposit films at high speed; for example, ordinary printing paper is coated with micrometre-thick layers of clay at a speed of tens of meters per second. However, to coat nanometre thin films at high speed, vacuum techniques are typically required, which increases the complexity of the process. Here, we report a simple wet chemical method for the high-speed coating of films with thicknesses at the nanometre level. This soap-film coating technique is based on forcing a substrate through a soap film that contains nanomaterials. Molecules and nanomaterials can be deposited at a thickness ranging from less than a monolayer to several layers at speeds up to meters per second. We believe that the soap-film coating method is potentially important for industrial-scale nanotechnology.

Applications of thin films^1^ can be found in many fields including microelectronics^2^, solar cells^3^, glass windows^4^ and consumer packaging^5^. Numerous techniques exist for the deposition of thin films^1^, including physical and chemical vapour deposition and wet chemical methods such as Langmuir–Blodgett^6^,^7^, dip-coating^8^, spin-coating^9^ or self-assembly^10^; however, all these techniques usually exhibit low deposition speeds compared to roll-to-roll (RoR) methods^2^,^5^,^11^. RoR have impressive speed of tens of m/s; however, the thicknesses of the deposited thin films are rarely less than 1 μm. One exception to this limitation is the more complex vacuum RoR techniques^12^, which are primarily used to produce thin (10–40 nm) aluminium coatings on plastic films for packaging applications^12^. These Al films are evaporated at speeds of up to 20 m/s, whereas for other RoR vacuum coatings, the speed is one or several orders of magnitude slower^12^.

Here, we demonstrate a new simple wet-chemical method for depositing nanometer thin films at a speed comparable to the one employed in the RoR techniques. This method, soap-film coating (SFC) is based on a simple concept: a soap film is created from a mixture of a surfactant solution and nanomaterials, and, when a substrate is forced through this film, a thin layer of nanomaterials is deposited onto the substrate (Fig. 1a). In addition to a single film, many soap bubbles could be used in the same manner (Fig. 1b), and we call this variant bubble soap-film coating (bSFC). The difference between the two variants is primarily a practical one, where the bSFC method might be easier to implement in an industrial setting because new bubbles could be continuously regenerated if they burst, which is not easily implemented in the single-film setting. For more details, see section ‘Methods’ below. The SFC method is useful in laboratory settings; in the simplest variant, it can be achieved by hand-coating, which allows an experiment to be set up in a matter of minutes (see Supplementary Section ‘Tutorial on simple soap-film coating’). We have primarily used the SFC method in this study to address the different physical parameters of the process.

A comparison of the SFC method with other wet chemical techniques (Fig. 1c) reveals that the primary advantage of SFC is the combination of its high coating speed and the ability to coat sub-micrometre thin layers, which is a combination that cannot be easily achieved using other wet chemical coating techniques and is important because this dual ability enables a number of applications to be addressed.

Another method related to SFC is the use of foam as a carrier phase, which is an established technology in the textile and nonwoven industries^13^ and is primarily used as a route to efficiently distribute dyestuffs or finishing agents while minimising the amount of required water. Previous efforts in the paper industry^14^,^15^,^16^ to utilise foam for coatings have been reported, and this application has recently garnered renewed interest^17^, such as in the foam coating of silica nanoparticles onto paper^17^. In this foam-coating technique, the entire foam is spread onto the substrate and allowed to dry, whereas in the SFC method, the soap film or bubbles are instead penetrated by the substrate. However, these methods are similar in that both techniques utilise foam as a carrier phase for molecules or particles. The single film SFC method does not appear to have attracted any previous attention, although previous works have described methods that resemble the SFC method, such as studies on attaching an entire soap bubble to a substrate^18^ or using polymer bubbles for the ordering of carbon nanotubes^19^; however, these methods cannot be easily implemented for large-scale coatings.

## Results

**Figure 2:**
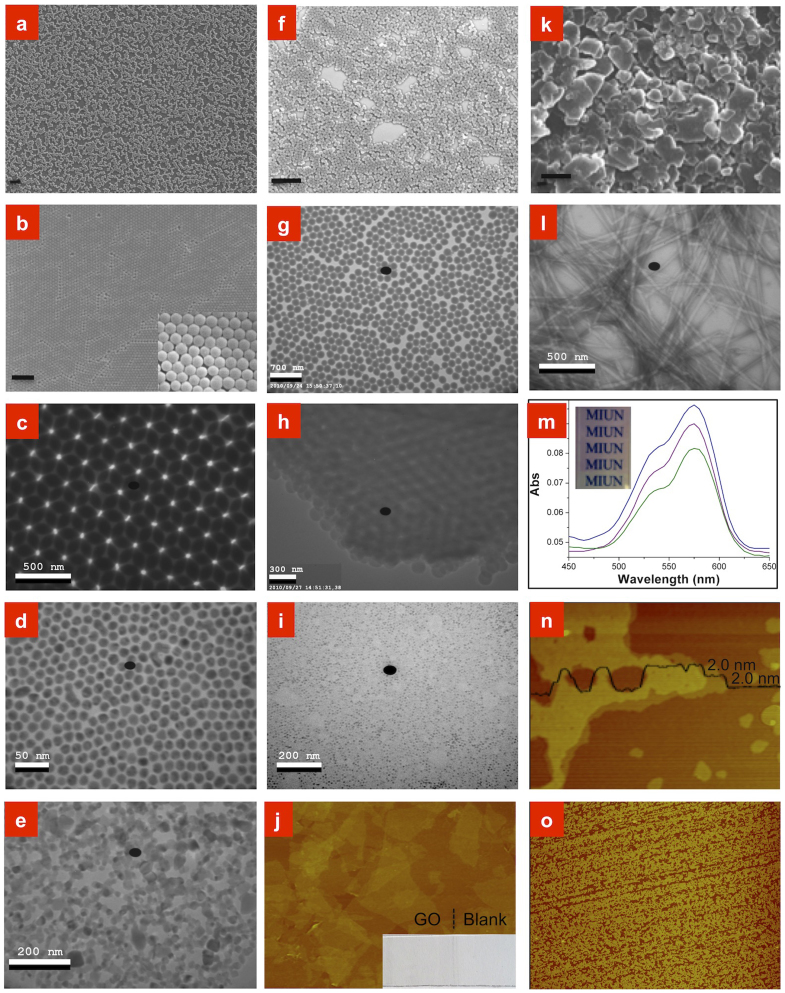
Soap-film coating of nanoparticles, layered materials, nanowires, and molecules. (a-e) Inorganic nanoparticles. (a–c) SFC-coated silica films with silica weight concentrations of 1.0 wt%, 2.1 wt% and 2.9 wt%. (a) scanning electron microscope (SEM) image of a 240-nm sub-monolayer of silica nanoparticles (scale bar is 2 μm). (b) SEM image of a 240 nm monolayer of silica nanoparticles (scale bar is 2 μm, and the inset shows an image at higher magnification). (c) Transmission electron microscope (TEM) image of a double-layer of silica nanoparticles. (The black dot in all the TEM images is an imaging artefact.) (d) TEM image of a monolayer of gold nanoparticles. (e) TEM image of a single-layer of TiO_2_ nanoparticles. (f–j) Organic and bio-organic nanoparticles. (f–h) SFC-coated polystyrene nanoparticles. See SEM images of triple- and multi-layers in Supplementary Fig. S10. (f) Sub-monolayer of polystyrene (scale bar is 2 μm). (g) Monolayer of polystyrene. (h) Triple-layer of polystyrene. (i) TEM image of a monolayer of ferritin. See higher magnification images in Supplementary Information. (j–o) Coated layered materials, nanowires, and molecules. (j) Atomic force microscope (AFM) image of a <1.5-layer GO film (3 μm × 2 μm). The inset shows an image of the coated layer after it was annealed at 240°C for 2 min. See transmittance measurements of this GO film in Supplementary Fig. S1. (k) SEM image of clay on glass (scale bar is 2 μm). (l) SFC-coated nanocellulose, also see the image in Supplementary Fig. S5. (m) Absorbance spectra of 1~3 times coated and an image of Rhodamine B on a glass slide. (n) and (o) Small-scale (2 μm × 1.5 μm) and large-scale (20 μm × 15 μm) AFM images of a SDS sub-double layer.

**Figure 3:**
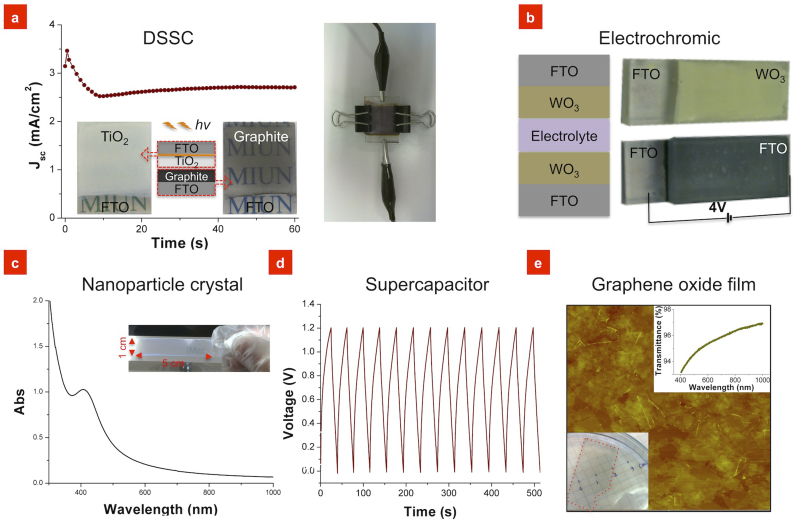
Examples of multilayers created using the soap-film coating method. (a) SFC-coated dye-sensitised solar cell (DSSC). The graph shows the initial current density when the DSSC was subjected to light. A maximum of 540 mV and 12 mA under 250 mW projector lamp were detected. The energy conversion rate was ~2.8% at a cell area of 0.38 cm^2^ and ~0.9% at 3.04 cm^2^. The inset shows the TiO_2_, the graphite-coated FTO glass, and the structure of the DSSC. The photo shows the assembled DSSC. (b) The structure of the electrochromic device and the colour of the WO_3_ film before and after a 4 V bias was applied to the FTO electrodes. (c) Nanoparticle crystal of 240 nm silica nanoparticles on a glass slide (10 coating cycles, 14 wt% particles, 8 cm/s), which exhibits an absorbance peak at 405 nm. The inset shows a photo of this crystal with a light-blue colour. These types of crystals might be explored for photonic crystal implementations. (d) Charge and discharge curves of a supercapacitor produced from SFC-coated 50 nm thick GO films, where the GO films were coated onto two silver foil electrodes and annealed at 400°C. (e) Free-floating graphene oxide (GO) film coated with the SFC method. The 5 μm × 5 μm AFM image shows a 2.6 nm thick GO film, which was the thinnest film that can be separated from the substrate without rupturing the film. The photo (inset, bottom left) shows the 2.6 nm thick GO film floating on the surface of an ammonia solution after being annealed at 240°C and separated from a silicon wafer. The film had a 95% transmittance at 550 nm (inset, top right).

Materials in the form of nanoparticles (Figs. 2a–i), layered nanomaterials (Figs. 2j, 2k), nanowires (Fig. 2l), or molecules (Fig. 2m–o), can all be deposited (for more figures, see Supplementary Information). Highly ordered packing of spherical nanoparticles for a number of materials, such as silica (Figs. 2a–c), gold (Fig. 2d), and polystyrene (Figs. 2f–h), are observed. These nanoparticle crystals were deposited at a relatively low speed of 8 cm/s; however, when the particle concentration was increased, a crystal still formed; for example, silica nanoparticles at a concentration of 11% produced an ordered monolayer at 1.2 m/s (Supplementary Fig. S6). Single- or multilayers of irregular particles, e.g., TiO_2_ (Fig. 2e), did not exhibit any ordering. However, for irregular layered materials, ordering was still observed because the layers were preferentially ordered along the surface direction (Figs. 2j, 2k); this phenomenon is not necessarily the case for other coating methods, such as doctor-blade coating (Supplementary Fig. S9). An interesting implication of this layered ordering is the SFC deposition of thin graphene oxide (GO) films with an area of several square centimetres (Fig. 2j). The SFC was independent of the material, and inorganic (non-metal, metal, metal oxide, and composites), organic (polystyrene), and biological materials (ferritin, Fig. 2i) could all be deposited. Water-soluble molecules were also deposited using the SFC method, such as the dye molecules shown in Fig. 2m and in Fig. 3d. A second class of molecules that are compatible with SFC is amphiphilic ones, as demonstrated in the deposition of a double layer of sodium dodecyl sulphate (SDS) (Figs. 2n, 2o) that had, as expected^20^, a thickness of 4.0 nm. This latter coating resembled that obtained with the Langmuir–Blodgett method, and the entire class of materials compatible with this method might subsequently be addressed using the SFC method, at least if a higher degree of defects is tolerable in the coated films.

To demonstrate the production of multilayers, we developed prototypes for some simple applications. The first multilayer prototype, a dye-sensitised solar cell (DSSC), is composed of five layers divided into two electrodes that consist of fluorine-doped tin oxide (FTO)/Au/TiO_2_ and graphite/FTO. Figure 3a shows the time dependency of the current density of a DSSC with an area of 3.04 cm^2^ under a projector lamp with a ~0.9% energy conversion rate (~2.8% for a 0.38 cm^2^ cell). A second application was an electrochromic device that was SFC-coated onto a FTO glass slide, and Fig. 3b shows the change in colour of a WO_3_ film (Supplementary Fig. S3) when biased at 4 V. A third prototype was nanoparticle crystals prepared from the previously described close-packed silica nanoparticles (Figs. 2a–c) with an absorbance band at 405 nm (Fig. 3c). Yet another application is a graphene-based supercapacitor constructed using the two previously described electrodes, a filter paper as a separator membrane, and KOH solution as an electrolyte, which resulted in a competitive^21^ capacitance of 130 F/g (Fig. 3d). Details on more multilayer SFC prototypes can be found in Supplementary Section ‘4 Multilayer applications’, including the free-floating GO film shown in Fig. 3e and the SFC layer-by-layer coatings of silica and the protein ferritin.

## Discussion

The different types of materials that were investigated (Fig. 2), to understand the limitations of the SFC method, showed that the method was quite general, from organic to inorganic materials may be used, as long as the particles mix or the molecules dissolve in the surfactant solution. Thus different types of material is compatible with the SFC method, but neither the shape is a limiting factor of the SFC method as materials in the form of nanoparticles layered nanomaterials, nanowires, or molecules, can all be deposited (Fig. 2). While the SFC method itself is not sensitive to the surface chemistry of the coating materials, there is potentially a problem in the choice of surfactant due to interactions between the surfactant and the nanoparticles. For the metal oxide nanoparticles we used a mixture of SDS and DOTAB instead of only SDS for other materials. The reason for this was that with only SDS the metal oxide particles aggregate to larger clusters and sediment, and with only DOTAB the soap film was too unstable for the SFC method.

**Figure 4:**
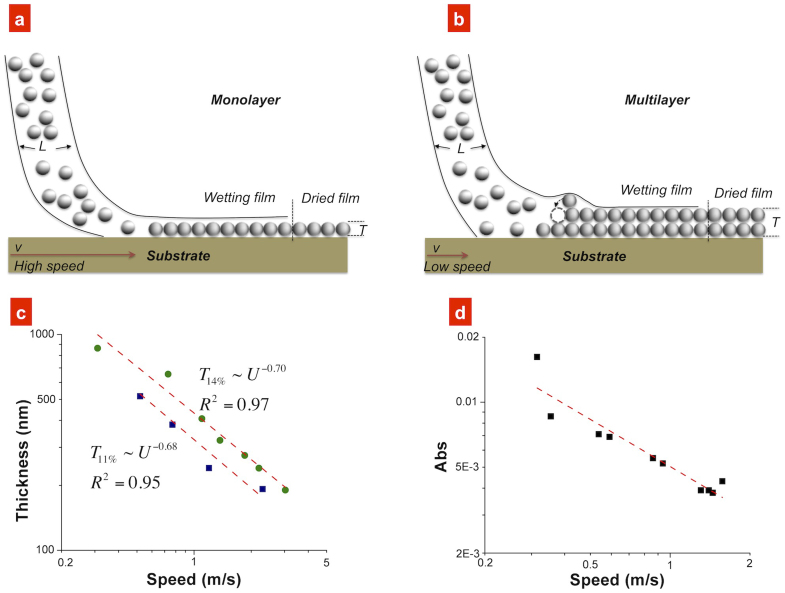
Thickness of films produced using soap-film coating. (a) Schematic drawing of the coating of a monolayer at high-speed and (b) a multilayer at a lower speed. (c) Thickness of a film of silica nanoparticles as a function of deposition speed (log–log diagram) for two different concentrations of nanoparticles - 11 wt% (square) and 14 wt% (circular). The measurements were performed using the SFC method; however, similar behaviours were observed using the bSFC method. (d) Optical absorption, which is proportional to the thickness, of methyl blue films produced using SFC. The dashed lines in (c) and (d) are least-squares fits to the data and indicate a thickness dependency of approximately ~*U*^−0.7^, where *U* is the speed of the substrate through the soap film.

The thickness of the film is determined by the speed of penetration into the soap film. During the deposition, the soap film was transferred onto the substrate and the particles were deposited while the water evaporated (Figs. 4a and b). A higher speed resulted in thinner films, which led to a practical upper speed limit for SFC of approximately 10 m/s (Figs. 4c–d); at higher speeds, the film will be too thin to be useful. Beyond this practical limit, the speed is likely controlled by external factors, which are common to other high-speed liquid coating problems, such as air entrancement^11^. We also note that the bSFC method will produce thicker films than SFC because several bubbles will be penetrated; however, the structure of the coated films was not altered between the two methods (Supplementary Fig. S8).

**Figure 5:**
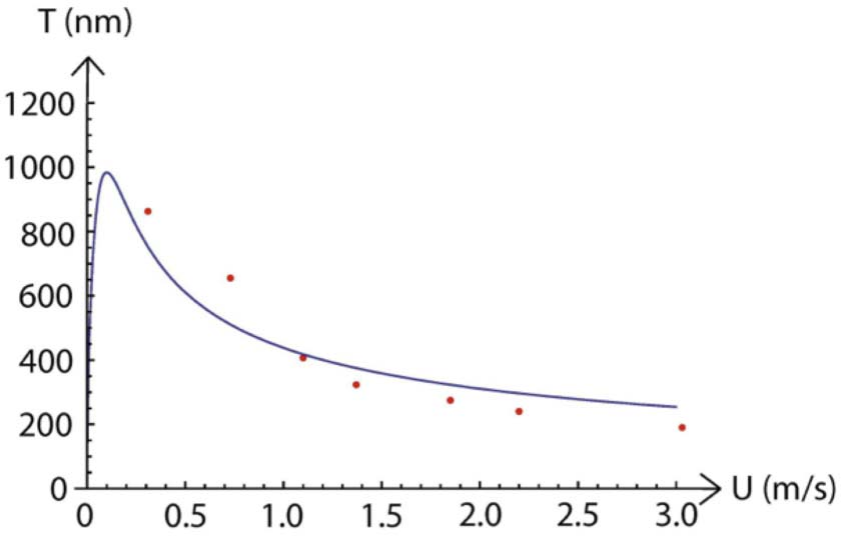
Speed versus thickness data (red dots) for the dried nanoparticle film. The blue solid line is from the Navier–Stokes based model, described in the text, fitted to the data points.

The coating thickness, *T*, was measured as a function of speed, *U*, and found to be *T ~U^A^*, where the exponent was *A* ≈ −0.7 for nanoparticles (Fig. 4c) and *A* ≈ −0.75 for the methyl blue (Fig. 4d). Two models were developed to explain this: a simple constant-flow model and one based on the Navier-Stokes equation, where the latter was inspired by a high-speed variant^22^ of dip-coating models^23^,^24^. These attempts gave, however, an exponent of −1 for the constant-flow and −0.5 for the Navier-Stokes model, which highlights the need for a more elaborate model. Fig. 5 shows the coated silica film thickness fit with the Navier-Stokes model, and it is obvious that at speed higher than 1 m/s the model gives too high values as compared with the experimental values, while for slower speed a too low. The reason is that the exponent of the speed U in the model is −0.5, whereas the experimental value is −0.7 (see more discussion in Supplementary Section ‘Thickness model’).

For some of the applications of SFC coated nanoparticles films, post-coating steps might be needed. Sintering is often a required step in the final treatment of nanoparticle films, particularly when the requirements of the films include high electrical conductivity. In both the DSSC and the electrochromic device, we used thermal sintering steps. However, the sintering is not necessarily a limitation for a roll-to-roll implementation of foam coatings because high-speed methods currently exist in which sintering is performed using electromagnetic radiation, such as in infrared heating, flash sintering^25^, or microwave irradiation^26^. To investigate this type of sintering, a film of ITO nanoparticles^27^ coated onto glass using the SFC method was laser irradiated, which resulted in a sheet resistance of 2.2 kΩ/sq (see Supplementary Fig. S14).

In summary, soap-film coating has been demonstrated to be a general method for the high-speed coating of ordered nanostructures in a laboratory setting; however, the large-scale industrial implementation of this method remains to be demonstrated, although the industrial oriented attempts using related foam coating systems^13^,^14^,^15^,^16^,^17^ indicate that this step might be straightforward. Therefore, we believe that the simplicity and generality of the soap-film coating method combined with the unique speed/thickness performance makes it a candidate for implementation in large-scale industrial applications of nanotechnology.

## Methods

### Materials

All chemicals and the nanoparticles (otherwise synthesized in our lab) were purchased from Sigma without further purification. Some parts and chemicals from a tool kit for DSSC testing, purchased from Arbor Scientific, was used.

#### Characterisation

The instruments we used for characterisations include TEM (JEOL 2000FX), SEM (EVO 50, ZEISS), AFM (Nanoscope IIIa), spectrophotometry (UV-1800, SHIMADZU), a Micromanipulator 1800 (Micromanipulator), and a Versalaser 200 (Universal Laser Systems) system.

#### Soap-film coating set-up

**Figure 6:**
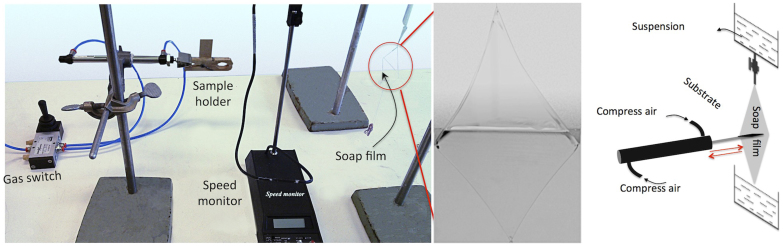
Soap-film coating setup. A substrate is fixed on a sample holder that can be moved forward or backward by controlling the compressed air at the two terminals of the cylinder. A speed monitor is placed between the sample holder and the soap-particle film to detect the speed of the moving substrate. The soap-particle film is held by a frame constructed from nylon wires, which is hanging in front of the sample holder. A “control wire” (see text) is placed on the soap film to increase the lifetime of the film. Above the frame, a funnel filled with suspensions that continuously refill the soap-particle film is mounted, as shown in the schematic drawing.

The coating setup (Fig. 6) is a homemade system that includes a compressed-air-controlled pneumatic cylinder, a clip for sample holding, and a prism-shaped frame constructed from nylon ties to hold the soap film or foam. The coating speed was controlled by changing the compressed-air pressure to the cylinder, which provided a speed range from cm/s to several m/s. A gas switch was used to change the direction of the piston. To achieve the lowest speed, we used two additional valves to further reduce the pressure.

To create a single soap film, a stick (we used iron wire) parallel to the frame can be moved from the top of the frame downward while the suspension flows. To increase the stability of the film, a nylon “control wire” was added after the film was created. The control wire was placed parallel to the table in the middle of the frame. The control wire guides the flow of the suspension into the area below the control wire, which was the effective area for penetration of the soap film by the substrate. This control wire guided a continuous refill of the effective area to avoid breaking the soap film due to consumption or evaporation during coating. Without the control wire, the suspension will not refill the centre of the film but will instead flow along the frame wire, which results in a shorter lifetime of the film. To create a bubble soap film, a single soap film was first produced, and air was subsequently injected through a valve on the funnel, which resulted in bubbles. The bubbles then flowed down and were held by the frame. When the bubble soap film was established, the substrate was coated by penetrating through the bubble films. For high-speed deposition (m/s), the resulting film is micrometre or nanometre thin, and the water will rapidly evaporate; however, when coating at lower speeds (cm/s), the thicker film will contain more water, and we sometimes used nitrogen gas to blow-dry the film.

#### Soap-film coating

The coating suspensions were prepared using double-distilled water, surfactant, and coating materials. The concentration of sodium dodecyl sulphate (SDS) was 0.6 wt% for the suspensions that only used SDS. For the metal oxide nanoparticles, identical amounts of SDS and dodecyltrimethylammonium bromide (DOTAB) were used with a total surfactant concentration of 0.6 wt%. Polystyrene nanoparticles were coated using the suspension after the synthetic process because the reaction produced surfactant. All substrates were rinsed with acid (H_2_SO_4_:HNO_3_ = 3:1), followed by acetone, ethanol and water. The substrates were dried with N_2_ gas and then fixed onto the coating equipment. The coating speed was controlled by adjusting the gas pressure on the pneumatic actuator (see Supplementary Information).

#### Synthesis of silica, polystyrene nanoparticles, and graphene oxide

Silica particles were synthesised using a wet chemical method^28^. Briefly, 9 ml of 25% ammonia was added to 50 ml of ethanol, which was followed by the addition of 0.3 ml of tetraethoxysilane (TEOS) while stirring. After 12 hours of reaction, the silica particles were collected by filtration and rinsed with ethanol and double-distilled water. The particles were then re-dispersed in double-distilled water.

Polystyrene particles were synthesised using the method described in reference^29^. First, a mixture of 25.0 ml of styrene, 2 ml of methacrylic acid, and 100 ml of water was heated to boiling. Then, 0.8 g of K_2_S_2_O_8_ was added as the initiator 5 minutes after the boiling started. The reaction was stopped after the mixture was stirred for approximately 1.5 hours.

Graphene oxide was synthesized from graphite by using modified Hummers^30^ method, and then was collected by filtration.

### Coating materials

#### Coating of silica particles

The synthesised silica particles were re-dispersed in double-distilled water with a weight concentration of 11 wt% or 14 wt%. Sodium dodecyl sulphate (SDS) was then added with a weight concentration of 0.6 wt%. The silica nanoparticles were SFC-coated onto silicon wafers at different speeds (Fig. 2c) to investigate the relationship between the coating speed and the thickness of the coated film. Silica nanoparticle concentrations of 1.0, 2.1, 2.9 and 5 wt% were used to investigate the influence of concentration.

#### Coating of polystyrene nanoparticles

To coat polystyrene nanoparticles, we simply used the suspension after the reaction without any surfactant added because the reaction produced some by-products that can serve as surfactants. The coating procedure was the sample as that described above. The coating speed was 8 cm/s. We note that this might be a more general approach as the usage of surfactant films and foams for the synthesis of nanoparticles is a well known technique^31^,^32^,^33^,^34^, making it straightforward to make soap film without additional surfactant.

#### Coating of graphene oxide (GO) films

We used a concentration of 0.17 wt% of graphene oxide (GO) and SFC-coated thin GO films onto silicon wafers or glass slides. The as-coated wet films on the substrates were gently dried using nitrogen gas. The coated substrates were then heated to 240°C for 2 min, where the colour of the film coated on the glass slide changed from transparent to light grey.

To create a 2.6 nm thick free-floating GO film, we coated a silicon wafer with two cycles at a concentration of 0.17 wt%. The film was then heated to 240°C, and the colour of the GO changed to grey. For further experiments and characterisation measurements, the GO was separated from the silicon wafer by being floated on an ammonia surface.

To produce electrodes for the supercapacitor study, we used a concentration of 0.3 wt% to SFC-coat thick GO films onto silver foils. The use of 10 coating cycles at a speed of 8 cm/s generated 50 nm thick films. The coated films were then annealed at 400°C for 10 min to reduce the GO.

#### Coating of TiO_2_ nanoparticles

TiO_2_ nanoparticles with an average diameter of 20 nm were dispersed in double-distilled water at a weight concentration of 3 wt% or 17 wt%. The lower concentration was used to coat a single layer of nanoparticles onto a carbon-coated copper grid that was used for TEM imaging, and the higher concentration was used to coat onto fluorine-doped tin oxide (FTO) glass to produce a dye-sensitised solar cell (DSSC). Note that we used a mixture of SDS (0.3 wt%) and dodecyltrimethylammonium bromide (DOTAB) (0.3 wt%) to create a well dispersed TiO_2_ suspension; otherwise, large aggregates of TiO_2_ nanoparticles appeared in the coated films. We used a speed of 8 cm/s for coating the TiO_2_ nanoparticles.

#### Coating of WO_3_ nanoparticles

WO_3_ nanoparticles with an average size of < 100 nm were purchased from Sigma-Aldrich. A suspension of 20 wt% WO_3_ nanoparticles was created using a mixture of SDS (0.3 wt%) and DOTAB (0.3 wt%) for performing the SFC coating. To create electrochromic electrodes, we coated WO_3_ nanoparticles onto a FTO glass substrate using 4 cycles at a speed of 8 cm/s. For TEM imaging of the WO_3_ nanoparticles, we coated the WO_3_ onto a copper grid at a concentration of 5 wt% (Fig. S3).

#### Coating of ferritin

To coat a protein, we used ferritin because of its good contrast during TEM imaging without the use of a stain (Fig. S4). Proteins can be foamed without the addition of a surfactant, and we attempted foaming both without surfactant and with 0.1 wt% SDS. When using SDS in the solution, the stability of the bubbles increased. The coating was performed on TEM copper grids. When the bubbles were formed, we penetrated the grid through the bubble or simply attached a grid to the bubble and broke the bubble with a needle. No obvious difference was observed during the TEM imaging between the samples that were coated with or without SDS.

#### Coating of rhodamine B and methyl blue

The weight concentrations of rhodamine B and methyl blue were 100 and 200 ppm, respectively. All films were coated onto glass slides for spectroscopic measurements.

#### Coating of cellulose

The weight concentration of nanocellulose used for coating was 2.8 wt%. The nanocellulose was sonicated in double-distilled water before SDS was added, which resulted in a more dispersed suspension that was suitable for SFC coating. The nanocellulose was coated onto a copper grid for imaging (Fig. S5).

#### Coating of SDS

SDS was coated onto silicon wafers at a weight concentration of 1.0 wt% with a coating speed of 8 cm/s.

#### Coating of clay

Clay was coated onto glass slides and paper at weight concentrations of 1.5 wt% for glass and 5% for paper.

#### Coating of gold nanoparticles

We used two methods to create gold nanoparticles for a suitable coating solution. These two methods are considerably more elaborate than simply mixing the particles with a SDS solution because the concentration of gold nanoparticles is too low to obtain a meaningful thickness of the particles, and the concentration of the particles had to subsequently be increased to reach a level where a close-packed film could be formed by SFC.

Method I. The gold nanoparticles were first modified with dodecanethiol according to the method of Bigioni et al.^35^, but in an aqueous solution. When the gold nanoparticles were modified, they floated on the solution surface. The careful injection of air below the floating gold nanoparticles resulted in the formation of bubbles with gold nanoparticles in the bubble film on the surface of the solution. Gold nanoparticles were coated onto a copper grid by attaching the grid to the bubbles.

Method II. We placed the gold nanoparticle solution under saturated toluene gas for 7 days^36^ to generate a gold nanoparticle film on the surface, which was followed by the addition of 1.0 wt% SDS. Then, the gold nanoparticles can be coated by penetrating a copper grid through a SFC lifted from the solution.

## Author Contributions

R.Y.Z. and H.O. conceived the soap-film coating method. R.Y.Z., M.H., B.A., S.F. and H.A. performed the experiments. M.A., H.E., P.E., N.J., H.E.N., M.N. and T.Ö. contributed to the experiments. M.O, S.E., T.U, K.K. and H.O. developed the coating model. R.Y.Z. and H.O. wrote the paper. All authors discussed the results and contributed to the manuscript.

## Supplementary Material

Supplementary Information Supplementary information

## Figures and Tables

**Figure 1:**
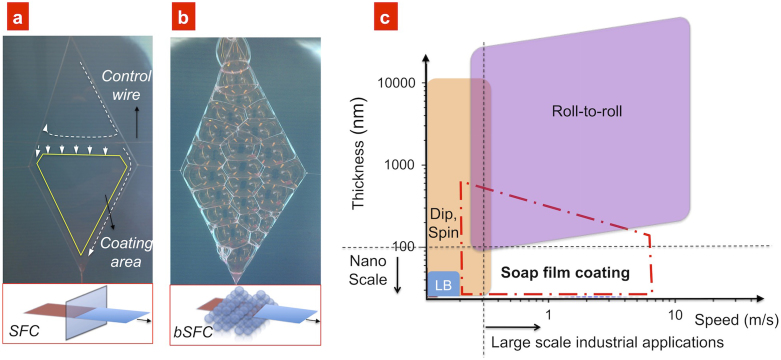
Soap-film coating method. (a) The principle of soap-film coating (SFC) and (b) bubble soap-film coating (bSFC). The single film and the foam are contained within a frame constructed from nylon wires. A “control wire” was added to increase the lifetime of the soap film by allowing a continuous refilling of liquid. (c) Comparison between some liquid coating methods. Several high-speed roll-to-roll techniques exist, such as doctor-blade coating and curtain coating; however, the thicknesses of the films are usually limited downwards. Wet chemical methods that can be used for the thinnest films include the molecular precision Langmuir–Blodgett (LB) technique^7^, dip^8^ and spin^9^ coating; however, these methods usually have a low deposition speed. The area that lacks high-speed and nano-thin deposition is addressed in this study using the soap-film coating method.
