# Supplementary material for: Soap-film coating: High-speed deposition of multilayer nanofilms
Source: Sci Rep. 2013 Mar 18;3:1477. doi: 10.1038/srep01477 (PMC3600599; doi:10.1038/srep01477)
Supplement: Supplementary Information [file srep01477-s1.pdf]

Supplementary information

Soap-film coating: High-speed deposition of multilayer nanofilms

Renyun Zhang, Henrik A. Andersson, Mattias Andersson, Britta Andres, Håkan Edlund, Per Edström, Sverker Edvardsson, Sven Forsberg, Magnus Hummelgård, Niklas Johansson, Kristoffer Karlsson, Hans-Erik Nilsson, Magnus Norgren, Martin Olsen, Tetsu Uesaka, Thomas Öhlund & Håkan Olin

Table of Contents

1. List of Figures **2**

2. Coating materials **3**

2.1. Graphene oxide *3*
2.2. WO_3 nanoparticles *4*
2.3. Ferritin *4*
2.4. Cellulose *5*

3. Coating processes **5**

3.1. Coating speed *5*
3.2. Coatings at different concentrations *6*
3.3. Comparison between SFC and bSFC *7*
3.4. Comparison between doctor-blade coating and SFC coating *8*
3.5. Coating of multilayers *8*

4. Multilayer applications **9**

4.1. Fabrication of a dye-sensitised solar cell (DSSC) *9*
4.2. Fabrication of an electrochromic device *9*
4.3. Fabrication of a supercapacitor *9*
4.4. Mixed-size nanoparticle coatings *9*
4.5. Free-floating graphene oxide film for making devices *11*
4.6. Sintering of an indium tin oxide (ITO) nanoparticle film *12*

5. Tutorial on simple soap-film coating **14**

6. Thickness models **15**

References **17**

1. List of Figures

Figure S1. Transmittance of a thin GO film (<1.5 layer).

Figure S2. AFM image of SFC-coated multilayer GO.

Figure S3. TEM image of SFC-coated WO_3 nanoparticles.

Figure S4. High magnification TEM image of SFC-coated ferritin.

Figure S5. Low magnification image of SFC-coated nanocellulose on a copper grid.

Figure S6. Silica films coated using different coating speeds.

Figure S7. Silica films coated from solutions with different concentrations.

Figure S8. Comparison of the SFC and bSFC methods.

Figure S9. Comparison of the effects of coating with a doctor blade and with SFC.

Figure S10. SEM images of multilayer polystyrene films.

Figure S11. Alternating-layer coating of silica nanoparticle and ferritin.

Figure S12. SFC-coated mixed sized silica nanoparticles.

Figure S13. Resistance changes of a 2.6 nm GO film that was annealed at 240°C.

Figure S14. Laser-sintered ITO film.

Figure S15. Tutorial of simple soap-film hand-coating procedure.

Figure S16. Constant-flow model.

Figure S17. Schematic drawing of the SFC Navier-Stokes model.

2. Coating materials

2.1. Graphene oxide

Here, the coating speed was 8 cm/s. The transmittance of the coated GO at 550 nm was 98.5%, and it was 96% after the coating was annealed at 240 °C (Fig. S1). This transmittance indicated a layer less than two layers in thickness[1], which was consistent with the AFM imaging described in the main text (Fig. 2n). An increase in the number of coating cycles resulted in thicker films, as shown in Fig. S2.

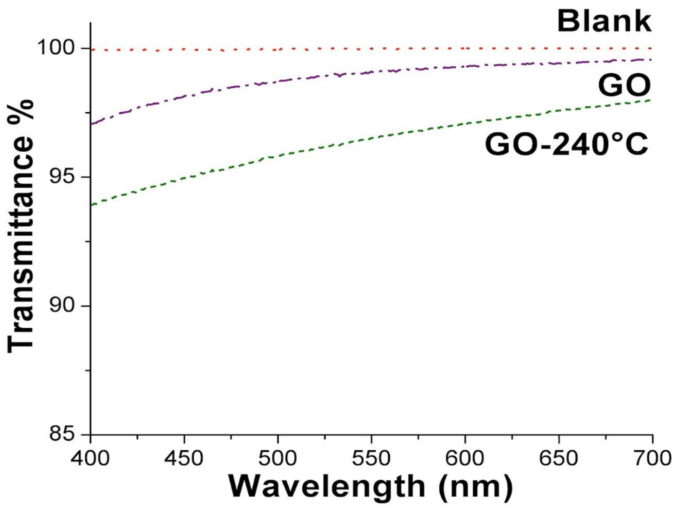

Figure S1. Transmittance of a thin GO film (<1.5 layers). This figure shows the transmittance of the GO film before and after being annealed at 240°C.

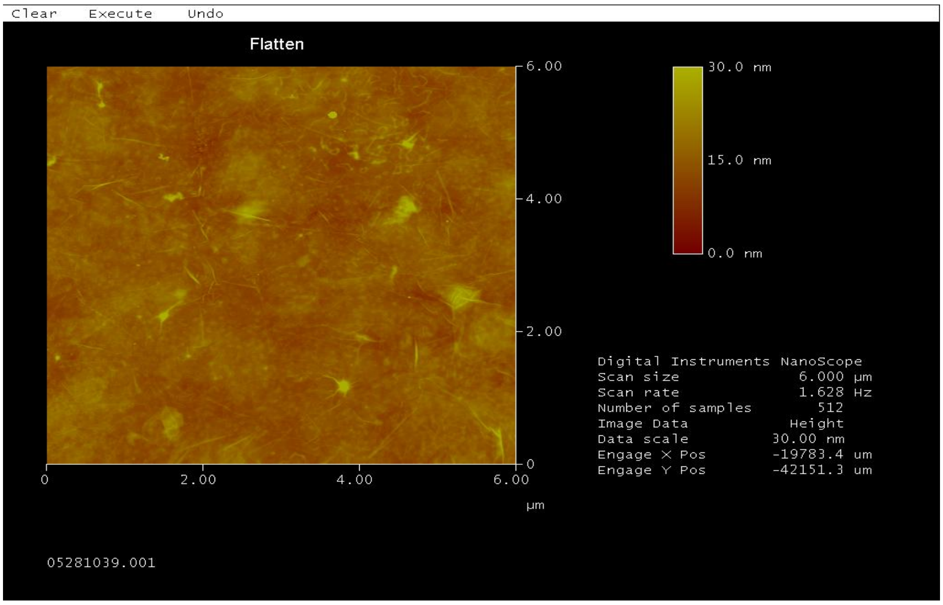

Figure S2. AFM image of SFC-coated multilayer GO. Concentration: 0.17 wt%, 8 cm/s coating speed, and six coating cycles.

2.2. WO_3 nanoparticles

For TEM imaging of the WO_3 nanoparticles, we coated the WO_3 onto a copper grid at a concentration of 5 wt%

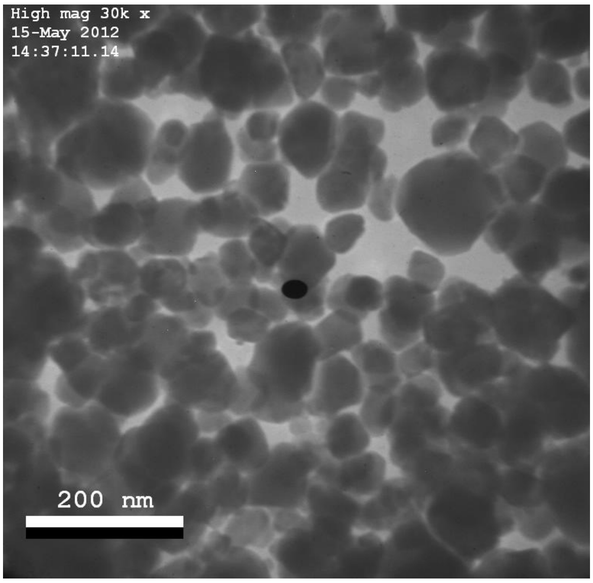

Figure S3. TEM image of SFC-coated WO_3 nanoparticles. The WO_3 nanoparticles were coated onto a copper grid for imaging. The black dot in the TEM image is an imaging artefact.

2.3. Ferritin

To coat a protein, we used ferritin because of its good contrast during TEM imaging without the use of a stain.

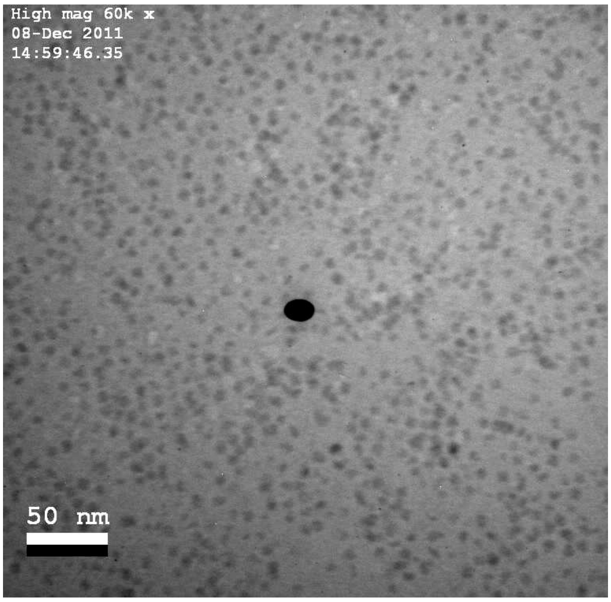

Figure S4. High-magnification TEM image of SFC-coated ferritin. This image shows a monolayer of ferritin using a copper grid as the substrate. The black dot in the TEM image is an imaging artefact.

2.4. Cellulose

The nanocellulose was coated onto a copper grid and stained with uranyl acetate for imaging.

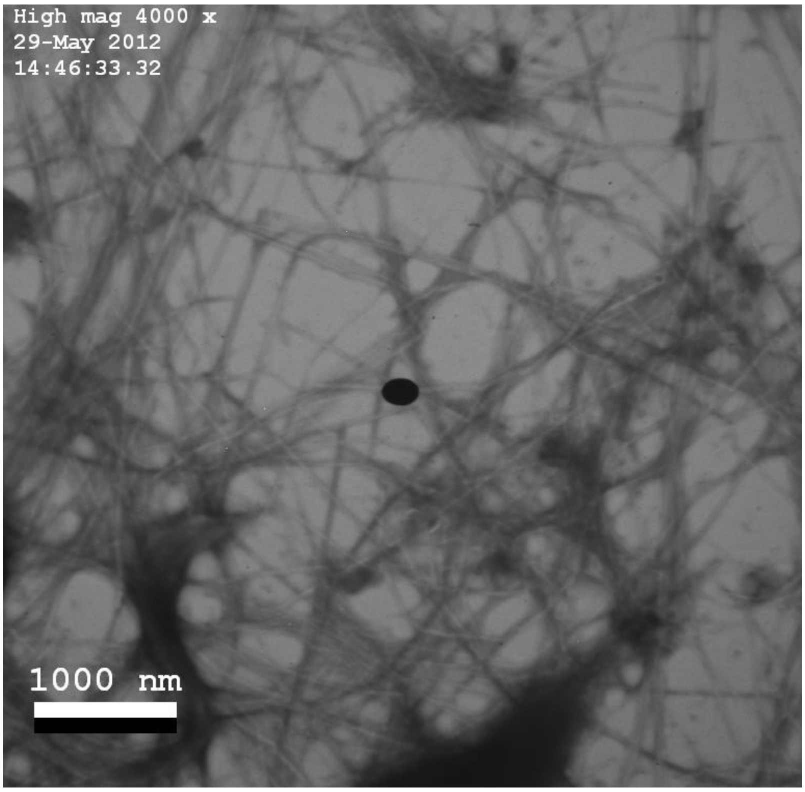

Figure S5. Low-magnification image of SFC-coated nanocellulose on a copper grid. The black dot in the TEM image is an imaging artefact.

3. Coating processes

3.1. Coating speed

To determine the relationship between the coating speed and the thickness of the film, we used two different materials: silica nanoparticles (diameter 240 nm) and methyl blue molecules. After the SFC coating process, the thickness of the film was measured by SEM imaging (Fig. S6 and Fig. 4c) for the nanoparticle films and by light absorption (Fig. 4d) for the methyl blue films using an absorbance spectrometer.

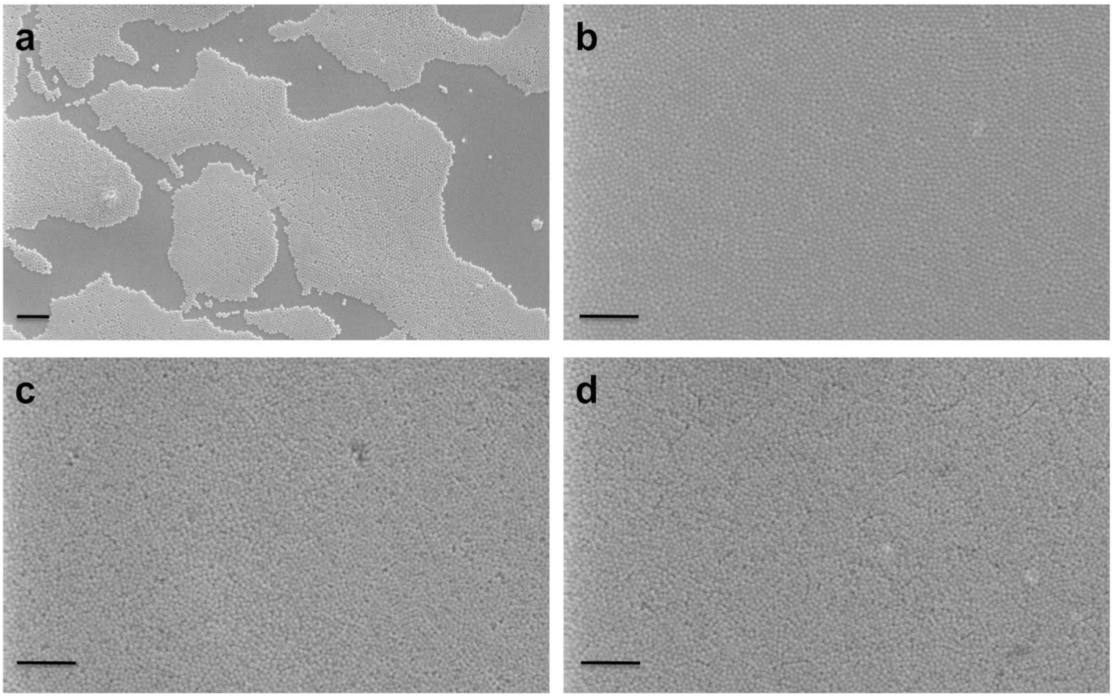

Figure S6. Silica films coated at different coating speeds: a) 2.3 m/s, b) 1.2 m/s, c) 0.77 m/s, and d) 0.52 m/s. Silica concentration: 11%. The scale bars represent 2 μm in all of the SEM images.

3.2. Coatings at different concentrations

The influence of concentration on the thickness of the film was determined via the coating of silica nanoparticles (Fig. S7). A sub-monolayer of silica nanoparticles (diameter of 240 nm) was coated, which resulted in an even distribution on the wafers (Figs. S7a and b). This even distribution was different from the sub-monolayer coated at higher speeds and with higher concentrations, as shown in Fig. S6. Figure S7c shows a large-area monolayer of silica nanoparticles; however, some defects are present. The results indicate that the monolayer coated at low speed (Fig. S7c) has better packing structures of nanoparticles than the one coated at high speed (Fig. S6b). Figure S7d shows a multilayer of silica nanoparticles produced using a coating solution with a silica concentration of 5.0 wt%.

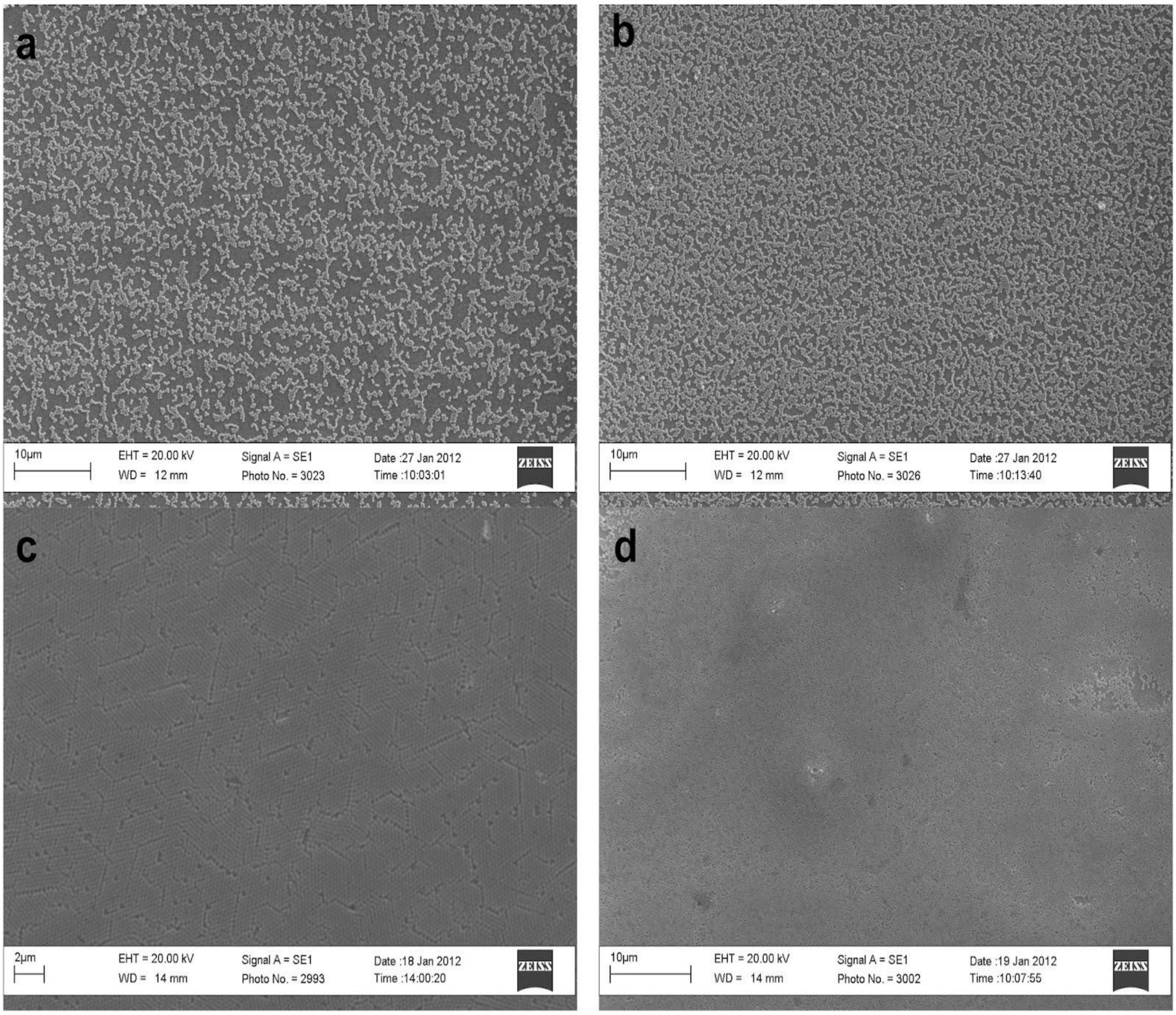

Figure S7. Silica film coated at different concentrations: a) 0.5%, b) 1.0 %, c) 2.1%, and d) 5%. Coating speed: 8 cm/s.

3.3. Comparison between SFC and bSFC

The surface structures of the coated multilayer silica nanoparticles deposited via SFC and bSFC exhibited no obvious differences (Fig. S8). However, the thickness of the bSFC-coated films using a single bubble layer, as shown in Fig. 1b in the main text, was thicker than those coated using SFC.

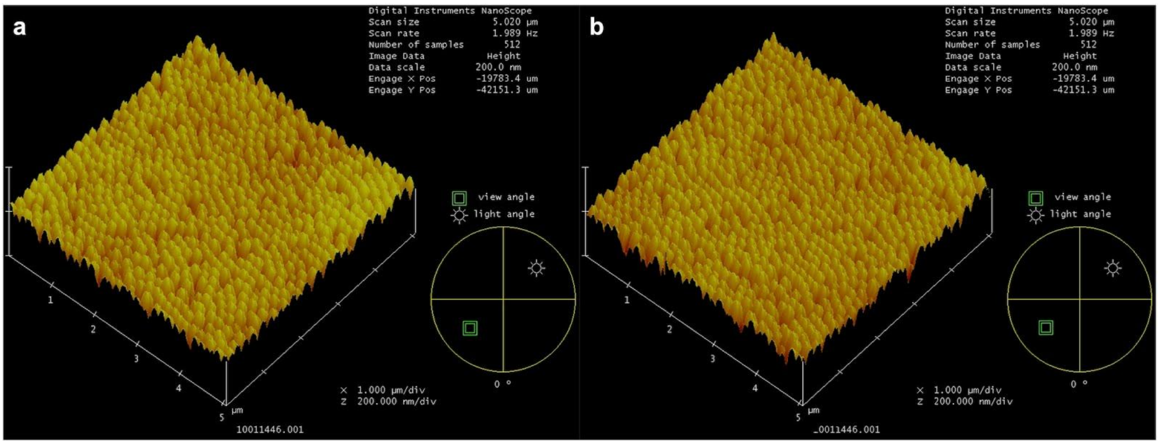

Figure S8. Comparison of the SFC and bSFC methods. AFM images of silica films on silicon wafers coated by **a,** the SFC method and **b,** the bSFM method.

3.4. Comparison between doctor-blade coating and SFC coating

Figure S9a shows the surface structure of clay on paper coated using the doctor-blade method, where we mixed water and clay and cast it onto paper using a blade. The SFC-coated surface (Fig. S9b) was smoother and exhibited a preferential ordering of the layered clay particles along the surface, whereas the doctor-blade-coated surface was rougher.

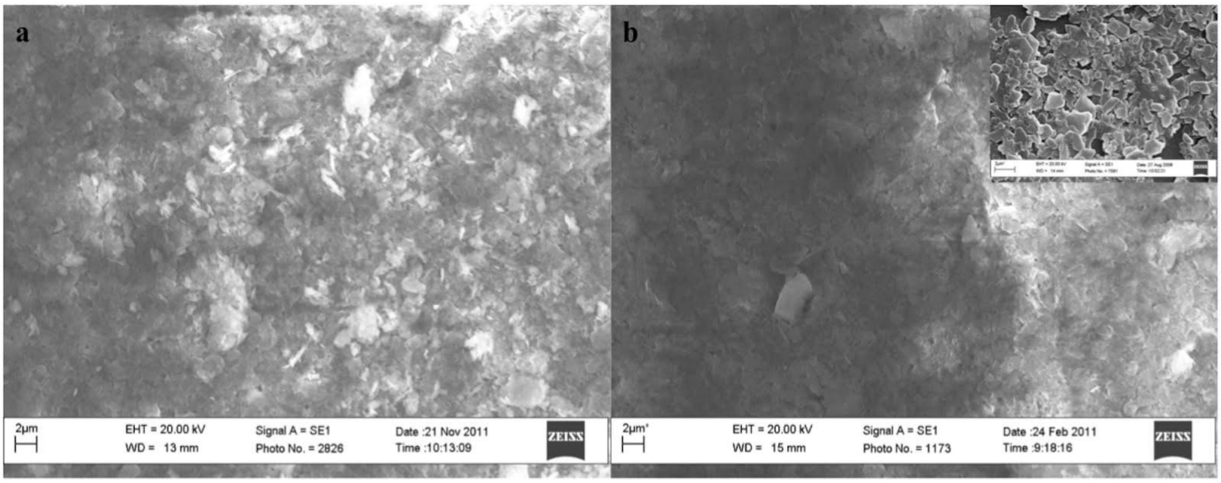

Figure S9. Comparison of the coating effect of doctor blade and SFC. a, SEM of doctor-blade-coated clay on paper. **b**, SEM of SFC-coated clay on paper.

3.5. Coating of multilayers

Figure S10a shows three layers of polystyrene nanoparticles coated by a repetition of three monolayer coating processes, which reveals control over the coating layers through repetition of the coating process. Figure S10b shows a multilayer of polystyrene nanoparticles.

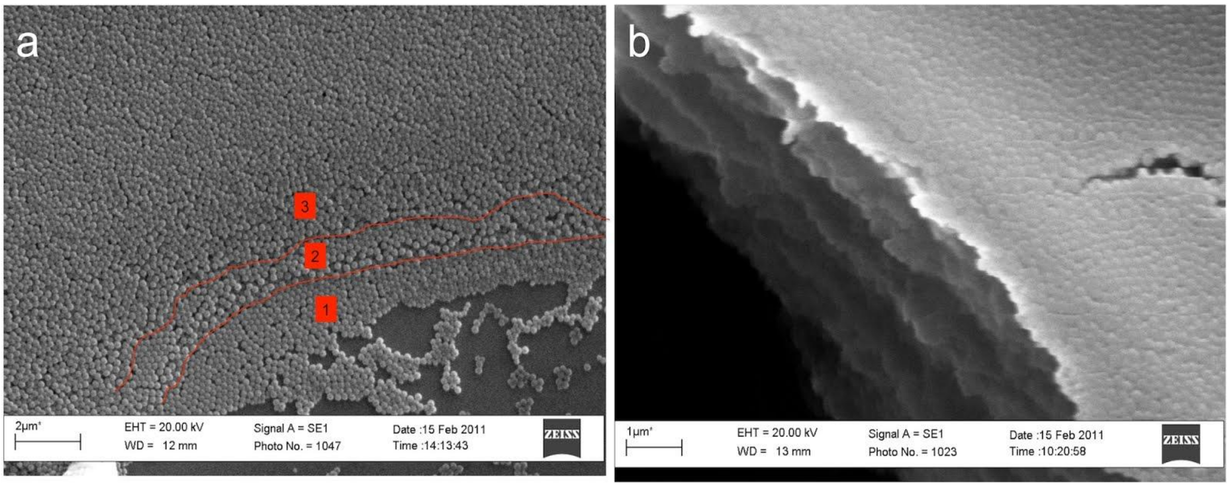

Figure S10. SEM images of multilayer polystyrene films: **a**, a triple layer of polystyrene and **b**, a multilayer of polystyrene nanoparticles.

4. Multilayer applications

4.1. Fabrication of a dye-sensitised solar cell (DSSC)

The DSSC was constructed using two FTO-coated glass slides. One of the slides was coated with graphite (concentration: 18%), and the other was first coated with a layer of porous gold nanofilm[2] and then with TiO_2 nanoparticles. The gold film was used to increase the conductivity. The DSSC tests were performed under direct sunlight and using a 250 W projector lamp. The light intensities were measured using a LUX meter.

4.2. Fabrication of an electrochromic device

Two FTO glass slides were SFC-coated using WO_3 nanoparticles (<100 nm, Sigma-Aldrich) and then simply assembled with two clips after the interface was filled with a 0.5 M H_2SO_4 solution as an electrolyte. A 4 V bias was applied to the FTO glass to change the colour. The coated WO_3 films were annealed at 400 °C for 30 min before use.

4.3. Fabrication of a supercapacitor

The supercapacitor was produced using two SFC-coated 50 nm GO films annealed at 400°C on silver foils as electrodes, a filter paper as a separator, and 9 M NaOH as an electrolyte[3]. The supercapacitor was charged and discharged at 1 mA to a maximum voltage of 1.2 V. The capacitance was calculated from the discharge curve of the fifth cycle.

4.4. Mixed-size nanoparticle coatings

We also alternately coated some films with particles of two sizes for layer-by-layer structures[4,5]. Two types of coatings were performed: 1) silica and a protein and 2) different-sized silica. In the first set of experiments, silica nanoparticles were coated first, followed by coating of the protein ferritin. By repeating the procedure, an alternating silica–ferritin structure was formed. The coating speed was 8 cm/s.

Figure S11 shows the alternating layer of silica nanoparticles and ferritin, where an ordered silica monolayer was first coated and then covered by another layer of ferritin. The figure shows that the ferritin layer was relatively even on the silica layer. The results suggest that the SFC coating method could be an efficient route to construct alternating-layer nanostructures.

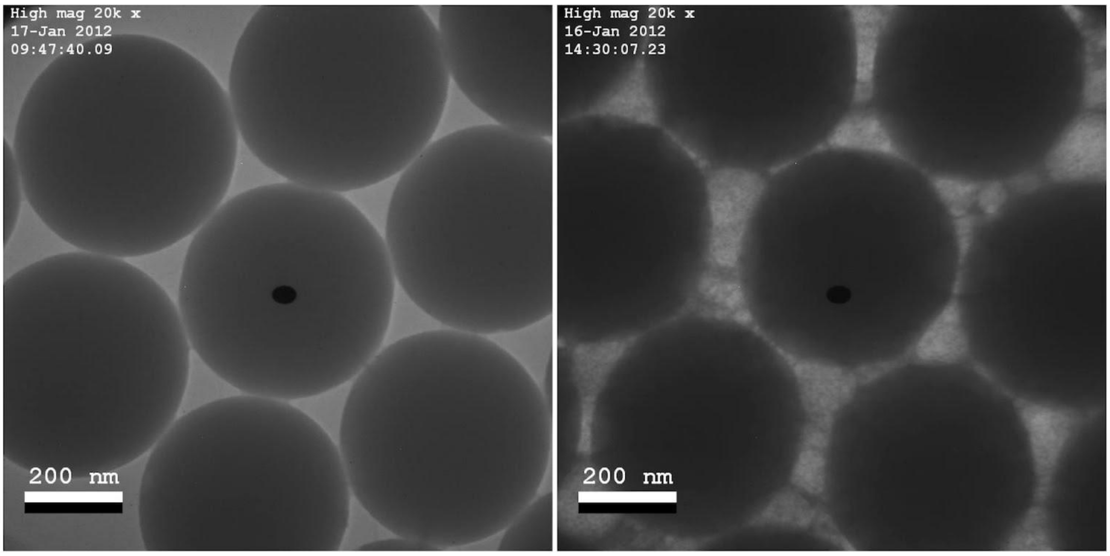

Figure S11. Alternating-layer coating of silica nanoparticles and ferritin. The silica particles were first coated at 8 cm/s at a concentration of 2.1% (left image). Then, ferritin was coated at a speed of 8 cm/s and at a concentration of 1.0 % (right image). The black dot in the TEM image is an imaging artefact.

The other type of mixed-particle-size coatings was created using silica nanoparticles of two different sizes with a concentration of 2.1 wt%. The larger-sized nanoparticles were first coated at a coating speed of 20 cm/s, and then the smaller-sized nanoparticles were coated at a coating speed of 30 cm/s. By repeating the procedure, a peculiar structure resulted, where the different-sized nanoparticles organised side-by-side (Fig. S12). When a larger area was viewed, the coated structures were observed to consist of numerous small islands on the wafer (see schematic in Fig. S12).

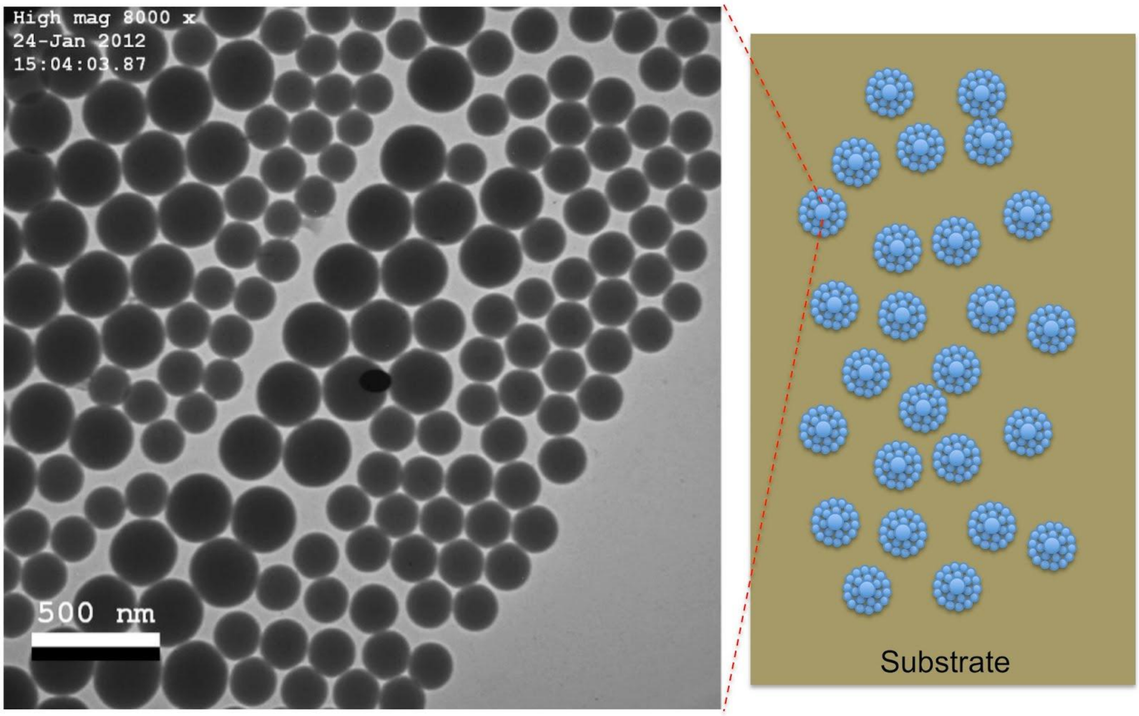

Figure S12. SFC-coated mixed sized silica nanoparticles. Large silica nanoparticles were first coated, followed by coating of the smaller ones. The image shows the result of two coating cycles. The black dot in the TEM image is an imaging artefact.

4.5. Free-floating graphene oxide film for making devices

A free-floating graphene oxide (GO) film is useful because it can be transferred to other substrates for further applications, e.g., in nanoelectronics. Here, we present a simple procedure for obtaining a free-floating GO film with a thickness as low as 2.6 nm, which corresponds to a double layer of GO.

The free GO film was produced by coating GO onto a silicon wafer followed by carefully dipping the coated wafer into ammonia. This dipping procedure separated the GO from the wafer. Notably, the GO film required annealing prior to this step because this process changed the colour to black, which allowed the GO film to be observed when it was floating on the ammonia; otherwise, determination of whether the film has been successfully separated is difficult. Figure 3e shows free-floating GO films; the thinnest film that could be floated as a whole film was 2.6 nm. The transmittance measurement of the thermally reduced film yielded a value of 95% at 550 nm (inset in Fig. 3e), which indicates a double-layer GO because the transmittance of individual graphene is ~2.3% (ref. 3). The I/V measurement exhibited linear behaviour.

We created a simple device to test the mechanical properties of the free, thermally annealed GO film. The coated film was first annealed at 240°C for 1 hour and then floated on ammonia. Subsequently, the film was transferred onto a PET film, and two electrodes (silver epoxy) were subsequently connected to the film (inset in Fig. S13). Mechanical tests revealed less than a 5% change in conductivity when the film was bent by 90°, which indicates good

mechanical stability (Fig. S13). This mechanical stability suggested that the free-floating film can be used as an electrode to create flexible electrodes.

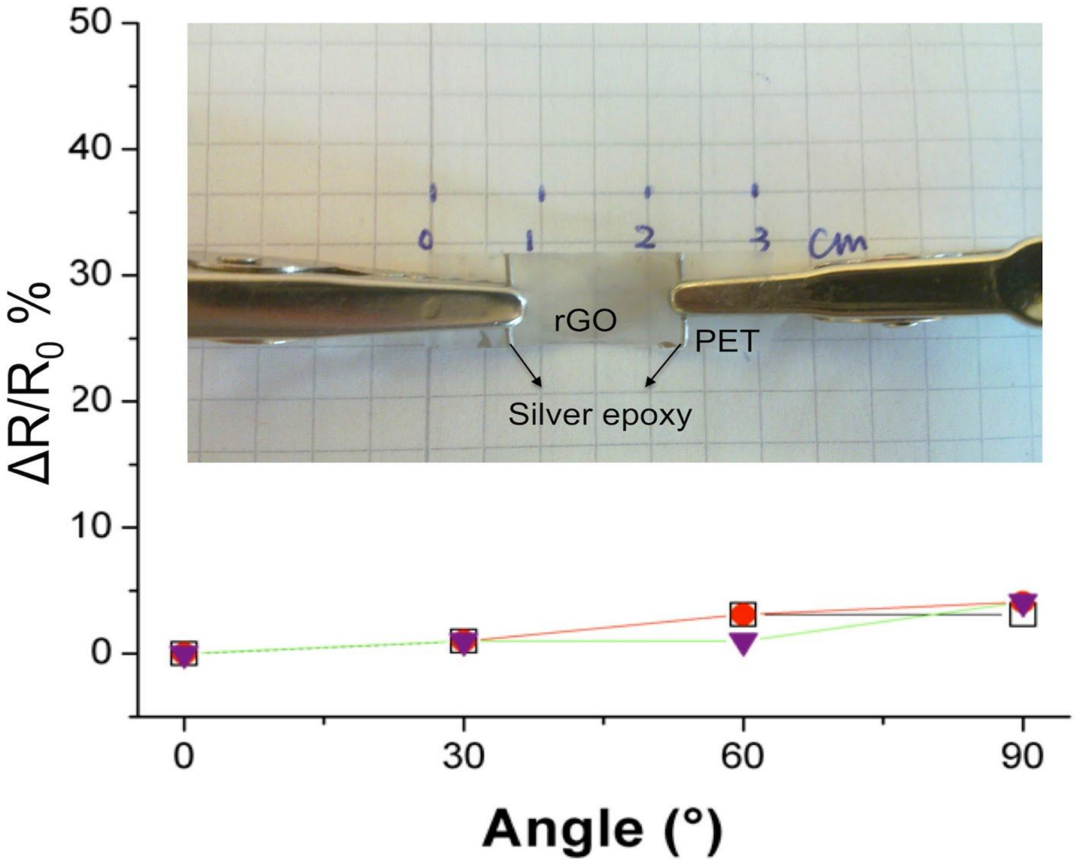

Figure S13. Resistance changes of a 2.6 nm GO film annealed at 240°C. This figure shows the resistance change at different bending angles, and the film exhibits less than a 5% change in resistance. The resistance did not show any obvious differences between three different tests, which indicates reversible behaviour.

4.6. Sintering of an indium tin oxide (ITO) nanoparticle film

Laser sintering can be used to sinter nanoparticles[6] and to consequently increase the conductivity of the film. Here, we sintered the SFC-coated ITO film on glass using a laser beam protected by nitrogen gas. The SFC-coated ITO nanoparticle film was sintered by a CO_2 laser using a 10 µm Versalaser 200 (Universal Laser Systems) system. The power of the laser was 10 % of the maximum power, which was 25 W. The average sheet resistance of the laser-sintered ITO films was measured to be 2.2 kΩ/sq. Figure S14 shows the transmittance of the sintered film and a photo of the sintered film on a glass slide.

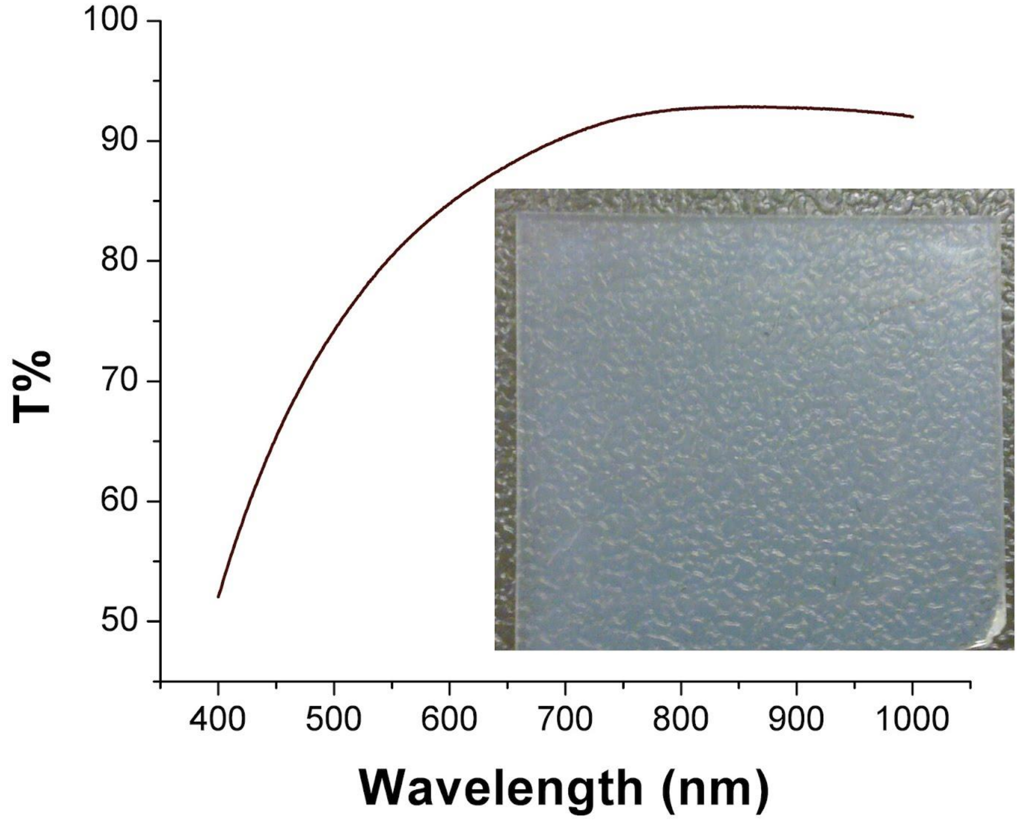

Figure S14. Laser-sintered ITO film. The figure shows the transmittance of the sintered film and a photo of a 2.5 × 2.5 cm^2 sintered film.

5. Tutorial on simple soap-film coating

A simple hand-coating SFC procedure can be used when no precise control over the film thickness is required. The protocol for this simple procedure is as follows:

1 Prepare a suspension of water, surfactant, and the material that is to be coated. The surfactant can be SDS, sodium lauryl sulphate (SLS), DOTAB or any other surfactant that can form a stable soap film.
2 Make a frame using, for example, iron wire or copper wire. The size of the frame should be larger than the cross-sectional area of the substrate that you plan to coat.
3 Dip the frame into the suspension, and then slowly lift it out. A thin film should be visible when the frame is lifted out of the suspension. Otherwise, repeat the procedure.
4 Penetrate the substrate through the soap film. Afterwards, you will see a film on the substrate. If you have several substrates to coat, coat each substrate the same amount of time after the film has been lifted from the suspension and by penetrating at the same place of the film, e.g., 1 cm from the top. The coating effect can be easily repeated using this procedure.
5 Dry the samples. You can dry the samples with a flow of air or nitrogen or allow them to dry naturally.

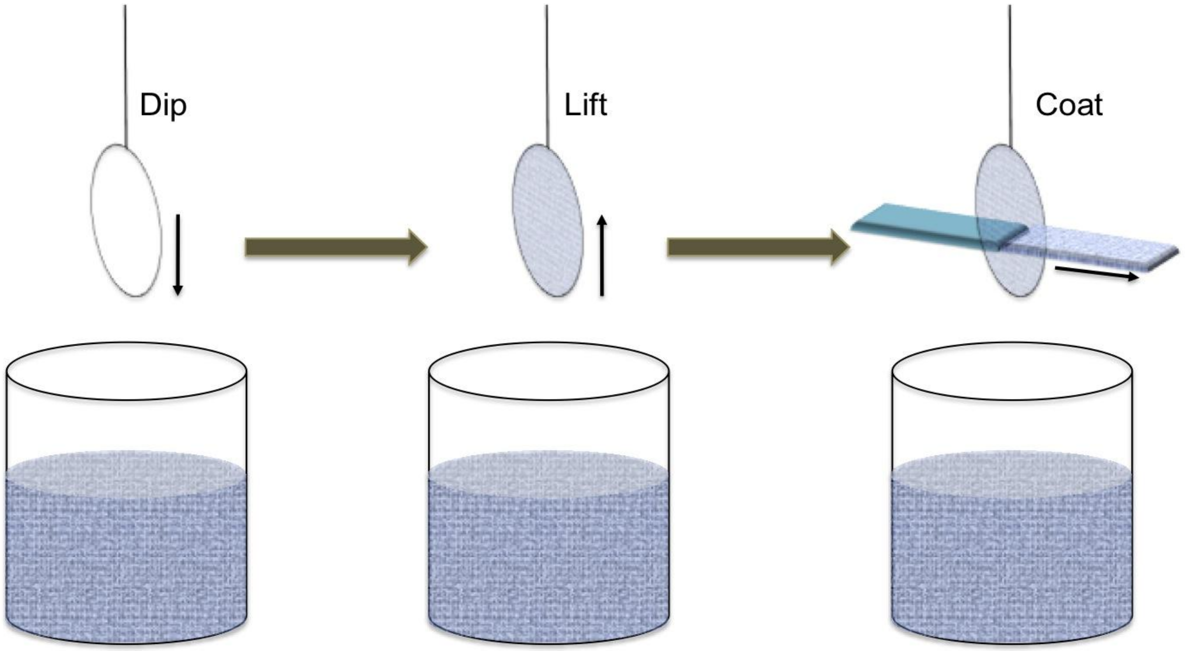

Figure S15. Tutorial of simple soap-film hand-coating procedure. A suspension of coating materials, water and surfactant needs to be prepared first. Then, create a frame from a metal wire, plastic plate, etc. To create a single soap film on the frame, simply dip the frame into the suspension and carefully lift up. Then, coat a substrate by penetrating it through the soap film.

6. Thickness models

Consider the following simple model, here called the constant-flow model, where $\phi\, dt$ is the soap-film volume that becomes deposited during the time dt and ϕ is the volumetric flow rate. This soap-film volume is assumed to become evenly distributed onto the surface $dA = wdx = wUdt$, where w is the width of the film and U is the speed of the substrate. The final volume of this film includes the thickness T as seen in Fig. S16. Thus we find that: $\phi\, dt = T\, dA = TwUdt$, which gives the following thickness relation $T = \phi/(wU)$. If one assumes a constant volumetric flow rate ϕ and width w, we note that the thickness T then has a simple $1/U$ dependence.

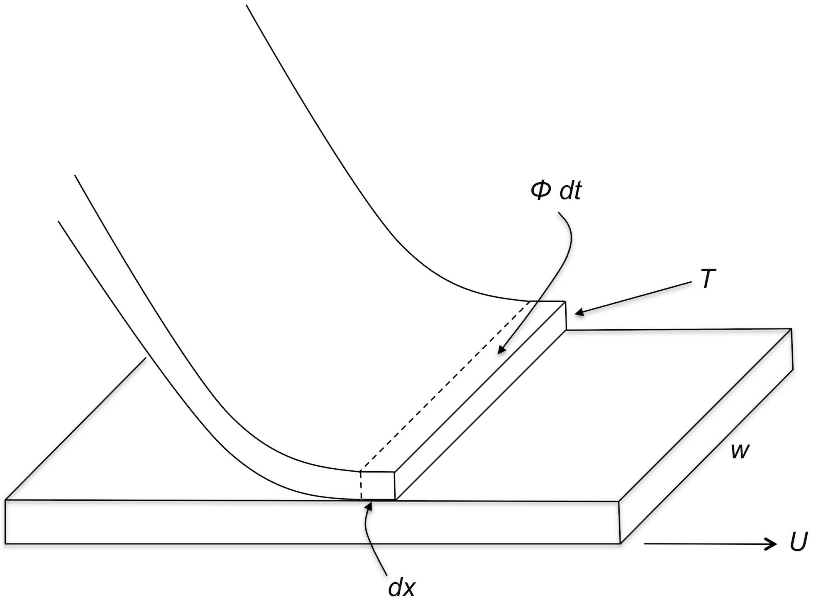

Figure S16. Constant-flow model. The soap-film is assumed to be deposited at a constant flow rate to the substrate that is moving with the speed U. The thickness T of the deposited film is given by $T \sim 1/U$ under the assumption that the volumetric flow rate f is constant.

A bit more elaborate approach is to apply fluid dynamical models. However, we cannot use the standard Landau–Levich–Derjaguin (LLD) dip-coating model for soap-film coating[7,8] because it is only valid for small capillary numbers, $Ca = \eta U/\gamma$, i.e., for low speeds (η is the viscosity, U the speed of the substrate, and γ the surface tension). Instead, below we develop a model inspired by a high-speed dip-coating model used for fibre coatings.[9] We can estimate the film thickness by modelling the coating thickness versus speed using the Navier–Stokes equation, which describes the flowing liquid:

$$\rho\, \partial\vec{v}/\partial t + \rho(\vec{v}\cdot\nabla)\vec{v} = -\nabla p + \eta\nabla^2\vec{v}.$$

At steady state, $\partial\vec{v}/\partial t = 0$. The pressure is lower where the film bends (Laplace pressure) relative to the film on the substrate by $p = -\gamma/\lambda$ holding back the flow (see Fig. S17). Assuming $T \ll L$, where T is the final film thickness on the plate and L is the soap film thickness, we can estimate the convective, pressure and viscous terms, respectively, in the Navier–Stokes equation as[10]

$$\rho(\vec{v}\cdot\nabla)\vec{v} \approx a\rho U^2/\lambda\, ;\, \nabla p \approx b\gamma/\lambda^2;\, \eta\nabla^2\vec{v} \approx c\eta U/h^2\, ;$$

where U is the speed of the substrate. The constants a, b, and c are of order unity. Note that ∇p is positive because the pressure is increasing in the flow direction. Under the distance λ (see Fig. S17), the speed is changing as it goes from almost zero in the film curtain to U in the film at the plate. The scale length for the static meniscus is $D^2 = \gamma/(\rho g)$, where g is the acceleration of gravity[8]. We assume $\lambda = D$. The change in the speed by the viscous shear force occurs over the distance h: the speed goes from U close to the plate to almost zero at the upper side of the film. In the viscous term, we have h squared because we are approximating a second derivative.

Note that the final thickness T of the fluid film when it is at rest relative to the plate is less than h because the volume flow should be the same in every cross-section. The ratio T/h depends on the velocity profile at the cross-section h (see Fig. S17) and is typically approximately ½. Using $h = 2T$, we find

$$h^2 = \frac{k_1 \eta \lambda}{\rho U}\left(\frac{1}{\left(1+\frac{k_2\gamma}{\lambda\rho U^2}\right)}\right) = (\frac{A^2}{U})/(1+\frac{B}{U^2})$$

where $k_1 = c/a$, and $k_2 = b/a$. The pressure term is consequently breaking, and the viscous term is driving. The thickness of the deposited films was measured to be roughly proportional to U to the power of −0.7, whereas the model predicts U to the power of −0.5.

The observed data for the nanoparticle film after the water has evaporated is shown in Fig. 5 in main text. We have the mass fraction of nanoparticles p_m in the fluid and the volume fraction p_V. These two fractions are related by

$$\frac{1}{p_v} = 1 + \frac{\left(\frac{1}{p_m} - 1\right)\rho_{SiO_2}}{\rho_{H_2O}}$$

where $\rho_{SiO2} = 2648$ kg/m^3 is the density of the silicon oxide nanoparticles, and ρ_{H2O} is the density of water. Using $p_m = 0.14$, as was used in one experiment, we calculate $p_V = 0.06$. Fitting the constants A and B to the data, we find that $A_{obs} = 7300$ nm (m/s)^{½} and $B_{obs} = 0.008$ m^2/s^2, where A has been corrected to the value before evaporation by dividing the dry film result by p_V.

However, we have $A^2 = k_1\eta\lambda/\rho$, $B = k_2\gamma/(\rho\lambda)$, and $\lambda^2 = \gamma/(\rho g)$. Using the constants $k_1 \approx 0.1$, $k_2 \approx 0.4$, dynamic viscosity $\eta = 0.001$ Ns/m^2, density $\rho = 1000$ kg/m^3 and surface tension $\gamma \approx 0.04$ N/m for soap water, we obtain agreement between the model and our observations.

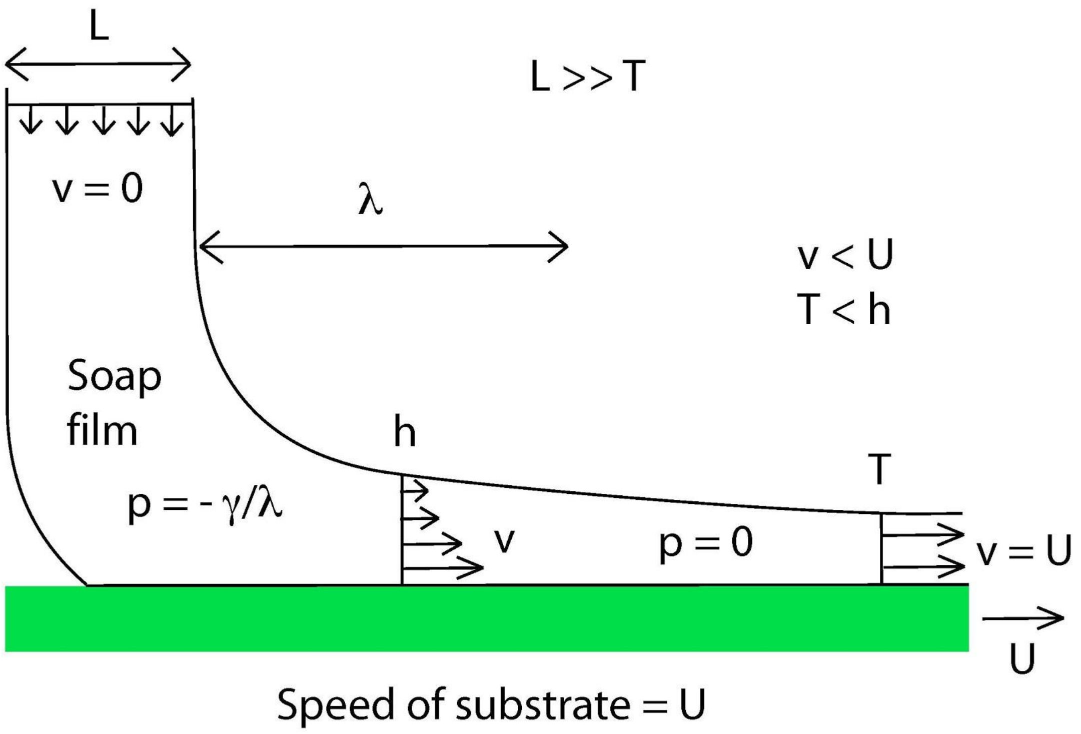

Figure S17. Schematic drawing of the SFC Navier-Stokes model. This figure shows the geometry of the film being dragged from a soap-film curtain onto a plate.

References

1 Nair, R. R. *et al.* Fine structure constant defines visual transparency of graphene. *Science* **320,** 1308-1308 (2008).

2 Zhang, R. Y., Hummergård, M., & Olin, H. Simple Fabrication of Gold Nanobelts and Patterns. *PLoS ONE* **7,** e30469 (2012).

3 Andres, B. *et al.* Supercapacitors with graphene coated paper electrodes. Nord. Pulp Pap. Res. J. **27,** 481-485 (2012).

4 Krogman, K. C., Lowery, J. L., Zacharia, N. S., Rutledge, G. C. & Hammond, P. T. Spraying asymmetry into functional membranes layer-by-layer. *Nat. Mater.* **8,** 512-518 (2009).

5 Luther, J. M. *et al.* Schottky solar cells based on colloidal nanocrystal films. *Nano Lett.* **8,** 3488-3492 (2008).

6 Ko, S. H. *et al.* All-inkjet-printed flexible electronics fabrication on a polymer substrate by low-temperature high-resolution selective laser sintering of metal nanoparticles. *Nanotechnology* **18,** 345202 (2007).

7 Landau, L. D. & Levich, V. G. Dragging of a liquid by a moving plate. Acta Physicochim. URSS **17,** 42-54 (1942).

8 Derjaguin B. V. On the thickness of the liquid film adhering to the walls of a vessel after emptying. Acta Physicochim. USSR **20,** 349-352 (1943).

9 de Ryck A. & Quéré D. Inertial coating of a fibre. *J. Fluid Mech.* **311,** 219-237 (1996).

10 Wilson S.D.R. The drag-out problem in film coating theory. *J. Eng. Math.* **16,** 209-221 (1982)
